# A Nationwide Survey of Australian General Practitioners on Antimicrobial Stewardship: Awareness, Uptake, Collaboration with Pharmacists and Improvement Strategies

**DOI:** 10.3390/antibiotics9060310

**Published:** 2020-06-08

**Authors:** Sajal K. Saha, David C. M. Kong, Karin Thursky, Danielle Mazza

**Affiliations:** 1 Department of General Practice, Monash University, Building 1, 270 Ferntree Gully Road, Notting Hill, Victoria 3168, Australia; Danielle.Mazza@monash.edu; 2 National Centre for Antimicrobial Stewardship (NCAS), The Peter Doherty Institute for Infection and Immunity, Melbourne, Victoria 3000, Australia; david.kong@monash.edu (D.C.M.K.); Karin.Thursky@mh.org.au (K.T.); 3 Centre for Medicine Use and Safety, Monash University, 381 Royal Parade Parkville, Victoria 3052, Australia; 4 Department of Medicine, University of Melbourne, Melbourne, Victoria 3010, Australia; 5 Pharmacy Department, Ballarat Health Services, Ballarat, Victoria 3350, Australia

**Keywords:** antimicrobial stewardship, general practitioners, GP–pharmacist collaboration, survey, primary care

## Abstract

Implementing antimicrobial stewardship (AMS) programs is central to optimise antimicrobial use in primary care. This study aims to assess general practitioners’ (GPs’) awareness of AMS, uptake of AMS strategies, attitudes towards GP–pharmacist collaboration in AMS and future AMS improvement strategies. A paper-based survey of nationally representative GPs across Australia was conducted in 2019. Of 386 respondent GPs, 68.9% were familiar with AMS. Respondents most frequently used the Therapeutic Guidelines (TG) (83.2%, 321/385) and delayed antimicrobial prescribing (72.2%, 278/385) strategies, whereas few utilised point-of-care tests (18.4%, 71/382), patient information leaflets (20.2%, 78/384), peer prescribing reports (15.5%, 60/384) and audit and feedback (9.8%, 38/384). GPs were receptive to pharmacists’ recommendations on the choice (50.5%, 192/381) and dose (63%, 241/382) of antimicrobials, and more than 60% (235/381) supported a policy fostering increased GP–pharmacist collaboration. Most GPs agreed to have AMS training (72%, 278/386), integration of electronic TG (eTG) with prescribing software (88.3%, 341/386) and policies limiting the prescribing of selected antimicrobials (74.4%, 287/386) in the future. Conclusively, GPs are aware of the importance of judicious antimicrobial prescribing but inadequately uptake evidence-based AMS strategies. The majority of GPs support GP–pharmacist collaborative AMS approaches to optimise antimicrobial use. Developing a feasible GP–pharmacist collaborative AMS implementation model and facilitating stewardship resources and training could foster AMS activities in primary care.

## 1. Introduction

Globally, optimising the use of antimicrobials in primary care is gaining much attention, with the awareness that most overuse of antimicrobials occurs in this setting [1]. Australia has been listed in the top 25% of countries in prescribing antibiotics in primary care [2]. General practitioners (GPs) in Australia prescribe antibiotics at much higher rates in acute rhinosinusitis (41% vs. 0.5–8%), acute otitis media (89% vs. 20–31%) and acute pharyngitis or tonsillitis (94% vs. 19–40%) when compared to the therapeutic guidelines (TG) [3]. A 2018 survey [4] of 572 antimicrobial prescriptions prescribed by GPs reported 57% of these prescriptions as inappropriate with problems of spectrum being too broad, dosing and duration. Therefore, researching antimicrobial stewardship (AMS) programs in Australian general practices is a priority.

AMS involves co-ordinated interventions or strategies to optimise antimicrobial use, ensure accessibility to effective antimicrobial therapy, improve patient outcomes and reduce antimicrobial resistance (AMR) [5]. In Australia, the National AMR Strategy 2015–2019 aimed to establish AMS programs in all health care settings [6], but the AMS clinical care standard is still not established in general practice [7]. AMS resources targeting Australian health professionals have been on the rise, such as indicators to support AMS, user guides and patient decision aids [8]. Furthermore, the National Prescribing Service’s (NPS) Medicine Wise programs has been supporting “Antibiotic Awareness Week”, antimicrobial prescribing courses, case studies and Continuous Professional Development (CPD) activities targeted at GPs [9,10,11]. However, participation of GPs in educational AMS programs and utilisation of existing stewardship resources is unclear.

Moreover, collaborative care models involving GPs and pharmacists can also support the implementation of AMS strategies [12]. A systematic review and meta-analysis [13] found that GP–pharmacist collaborative AMS strategies, such as group meetings, prescription audit and feedback, delayed-prescribing, academic detailing, stewardship education and training, are effective in reducing antibiotic prescribing (by up to 12%) and increasing guideline-adherent prescribing (by up to 16%) by GPs. Using point-of-care tests in a GP–community pharmacist (CP) collaborative model [14,15] has the potential to reduce the inappropriate use of antibiotics in treating influenza or pharyngitis. Furthermore, using shared decision-making approaches and patient information leaflets have also shown effective to minimise antibiotic use related to patient expectations [16]. To date, a thorough analysis of current implementation of these strategies in Australian general practices and GPs’ perceptions to collaboratively working with pharmacists as a team in AMS is lacking.

This study aimed to unpack GPs’ awareness of AMS, uptake of evidence-based AMS strategies and their attitudes towards GP–pharmacist collaborative approaches and other improvement strategies to optimise antimicrobial use in Australian primary care.

## 2. Results and Discussion

### 2.1. Literature Search

A systematic search strategy resulted in 568 articles from medical databases and 16 by manual search (Figure S1). After screening the titles and abstracts, the full texts of the remaining 41 articles were reviewed. Six AMS survey articles met the inclusion criteria and were used for extracting survey questions.

### 2.2. Response and Reliability

Of the 2500 GPs reached, 386 GPs responded, giving a response rate of 15.4% for the completed survey. Respondent’s characteristics are presented in Table 1. The distribution of surveyed GPs by gender, and states and territories was similar to the Australian census report 2017 (Table 1). Our data did not pass normality when assessed using Shapiro-Wilk test. The survey tool demonstrated good reliability (0.8 ≤ α < 0.9) with 0.837.

### 2.3. Demographic Characteristics

GPs’ response rates were proportionate to the workforce distribution of GPs among six states and two territories in Australia (Table 1). GPs who participated were from metro (60.9%), regional (19.2%), rural (16.1%) and remote (3.6%) locations. Seventy-nine percent of surveyed GPs held a Bachelor of Medicine and Bachelor of Surgery (MBBS) degree and 83.4% had more than 10 years of experiences practising in general practice. Two-thirds of GPs completed their medical training in Australia. More than a quarter of GPs (27.4%, 105/383) completed the NPS antimicrobial prescribing courses, although about a half (52.2%, 200/383) did not and one fifth (20.3%, 78/383) were not aware of the courses.

### 2.4. Awareness of AMS

**Figure 1:**
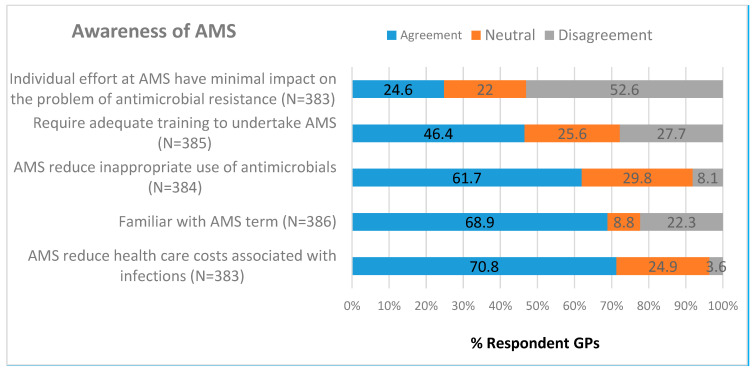
Proportion of GPs agreeing with the objectives and awareness of antimicrobial stewardship (AMS).

Figure 1 and Table S1 show the awareness of AMS. Most GPs (68.9%, 266/386) were familiar with AMS. GPs positively perceived the objectives of AMS that AMS programs reduce inappropriate use of antimicrobials (61.7%, 237/384) and health care costs (70.8%, 273/383). More than half of GPs (52.6%, 204/383) disagreed that their individual effort at AMS has minimal impact on reducing resistance. Approximately half of GPs (46.4%, 179/385) perceived that they require adequate training to undertake AMS. Overall, GPs demonstrated positive perceived awareness about AMS (median 4.0, IQR 1) (Table S1). Female GPs (*p* = 0.001) and GPs who completed the NPS antimicrobial prescribing courses (*p* = 0.000) had showed increased AMS awareness (Table S5 and S6).

### 2.5. Uptake of AMS Strategies

**Figure 2:**
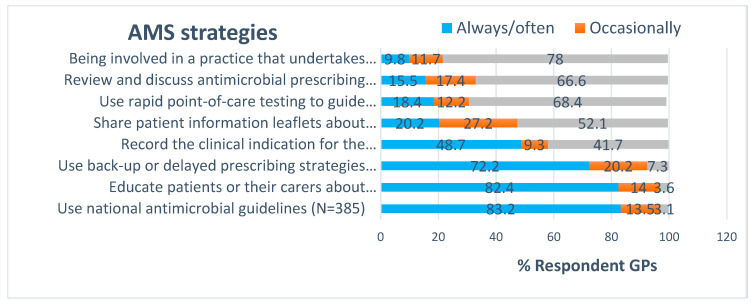
Proportion of GPs agreeing to uptake of AMS strategies when prescribing antimicrobials.

The use of AMS strategies by GPs is shown in Figure 2 and Table S2. Approximately half (51.3%, 198/385) often used the national antimicrobial guidelines when considering how to treat common infections, and only one-third (31.9%, 123/385) reported always using guidelines. Most GPs used delayed antimicrobial prescribing strategy where appropriate (always, [52.3%, 201/385] and often, [20%, 77/385]). Most respondents (82.4%, 316/383) always or often educated patients about the unintended consequences of antimicrobial use (e.g., resistance, effect on gut microbiota).

In contrast, only 20.2% (78/384) reported that they always or often shared patient information leaflets during counselling of patients who required antimicrobials or had infections. The use of point-of-care tests to confirm pharyngitis or flu was very low (18.4%, 71/382). Less than half (48.7%, 188/385) of respondents recorded the clinical indication for antimicrobials prescribed. Nearly 10% (38/384) of respondents had had their antimicrobial prescriptions audited and had received feedback. Few GPs (15.5%, 60/384) discussed antimicrobial prescribing of their practice with peer prescribers at least once a year. The overall median score 2.0 (IQR 1) reflected GPs’ poor-uptake of evidence-based AMS strategies (Table S2). GPs who had completed the NPS’ antimicrobial courses had increased (*p* = 0.000) uptake of AMS strategies (Table S5 and S6). Holding a MBBS degree was also found to be associated (*p* <0.014) with increased use of AMS strategies during a patient consultation (Table S5). According to the results of factor analysis (Figure S2 and Table S7), the adoption of AMS strategies during antimicrobial prescribing would depend on GPs’ personal behaviour or attitudes (component A) and the availability of AMS resources or structures (component B).

### 2.6. Attitudes Towards GP–Pharmacist Collaboration in AMS

**Figure 3:**
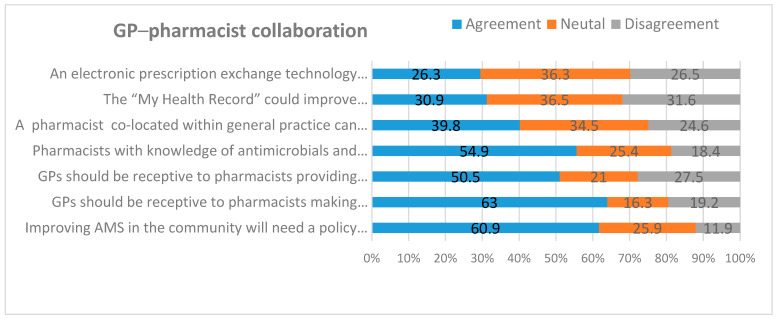
Proportion of GPs agreeing with GP–pharmacist collaborative approaches to improve AMS.

Figure 3 and Table S3 depict GP’s attitudes towards GP–pharmacist collaboration in AMS. More than 60% (235/381) of GPs supported a policy that would facilitate better collaborations between general practice and community pharmacy. Respondent GPs were receptive to pharmacists’ recommendations on the choice (50.5%, 192/381) and dose (63%, 241/382) of antimicrobials. More than half of GPs (55%, 212/385) agreed that a pharmacist with knowledge of antimicrobials and infections should attend regular group meetings of GPs to discuss antimicrobial pharmacotherapy. Although one-third of GPs (34.5%, 133/382) were unsure, 39.8% of GPs (152/382) were positive about the impact of pharmacist’s co-location in general practice to optimise antimicrobial therapy. A mixed response was amplified in the potential role of “My Health Record” in improving the communication between GPs and CPs about antimicrobial prescriptions. Overall, GPs showed equivocal attitudes towards GP–pharmacist collaboration in implementing AMS, but the majority of GPs supported a positive inter-professional collaboration. 

### 2.7. Attitudes Towards Future AMS Strategies

**Figure 4:**
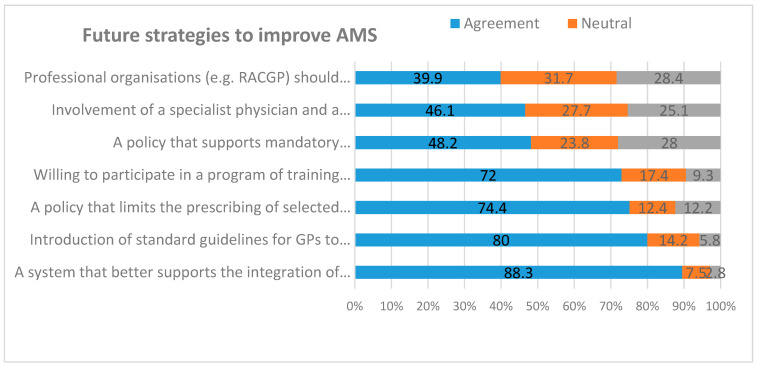
Proportion of GPs agreeing with future strategies to improve AMS in general practice. [RACGP, The Royal Australian College of General Practitioners].

Figure 4 and Table S4 demonstrate the attitudes of GPs towards future AMS strategies. Most were willing to participate in future AMS training (72%, 278/386), strongly supported the introduction of AMS guidelines (80%, 309/386) and better integration of eTG with their prescribing software (88.3%, 341/386). Three-quarters of respondents (74.4%, 287/386) supported a policy that would limit the prescribing of selected antimicrobials for certain clinical conditions. In contrast, nearly half (48.2%, 186/386) supported a policy that would mandate the documentation of clinical indication for prescribing antimicrobials.

Sixty percent of GPs were unsure or disagreed that professional organisations (e.g., The Royal Australian College of General Practitioners (RACGP) should define their roles in AMS. Less than half (46.1%, 178/386) supported the involvement of a specialist physician or a pharmacist to provide GPs with individualised antimicrobial prescribing advice and feedback. Overall, GPs were highly receptive to future AMS strategies, including education and training that would help them to optimise antimicrobial prescribing.

### 2.8. Barriers and Facilitators to Improve AMS

Of the surveyed participants, 57.25% of GPs (221/386) reported barriers and 41.5% of GPs (160/386) reported facilitators to implement AMS. Findings are summarised in Table 2.

#### 2.8.1. Barriers to Conducting AMS by GPs

The common barriers that GPs pointed out were related to patient, guidelines, organizational environment and structures, resources, technology and finance. GPs felt that the patient (e.g., expectations, desire for a quick-recovery, lack of awareness on antibiotic use risks and a patient’s late presentation with severe symptoms), using guidelines (e.g., cost, broad-recommendations and trustworthiness of guideline recommendations for some clinical conditions), lack of time, available AMS undertaking specific guidelines and their accessibility to ID physicians, pharmacists and microbiological services were common barriers to undertake AMS. Several respondents described CPs as just “dispensers” and that CPs do not have adequate knowledge to educate GPs in matters related to AMS. Many GPs were concerned about an inadequate understanding of a patient’s conditions by CPs to comment on their antimicrobial prescriptions. 

#### 2.8.2. Facilitators to Conducting AMS 

GPs’ suggested interventions to accelerate AMS activities focused on tools and technology (e.g., eTG, point-of-care tests, and clear guidelines on the AMS task), the person (e.g., GPs’ willingness to follow AMS guidance and training), organization (e.g., AMS training programs, accessibility to ID physicians, microbiologists and pharmacists, weekly practice meeting on AMS, audits and feedback, NPS visits and academic detailing), tasks (e.g., spending time on educating patients and delayed prescribing), the physical environment (e.g., patient education leaflets and posters and NPS hand-outs for treating infections) and the external environment (media campaigns, GP–pharmacist group meetings, GP–pharmacy practice agreement, policy limiting antibiotic use in particular indications and incentives for a longer consultation). Some GPs believed that effective collaboration could occur where GPs are willing to seek and accept the advice of pharmacists and pharmacologists regarding antimicrobial prescriptions; GPs then make an informed decision regarding the patient’s antimicrobial therapy, considering the patient’s condition and individual circumstances.

### 2.9. Discussions

This study, which, to our knowledge, is the largest study of its kind in Australia, found that most of the participating GPs were aware of AMS, and before prescribing antimicrobials, they used the Therapeutic Guidelines and delayed antibiotic prescribing strategies. However, the routine adoption of point-of-care tests, patient information leaflets, peer-prescribing reports and audit-feedback strategies was poor, probably due to a lack of AMS resources and structures. The majority of GPs would be receptive to pharmacists’ recommendations regarding the choice and dose of antimicrobials and policies fostering increased GP–pharmacist collaboration in AMS.

Although GPs were familiar with AMS, it is concerning that approximately half of the respondents still did not firmly believe that their individual efforts have the potential to reduce AMR. Pleasingly, most of the respondents were willing to undergo AMS education and training. Training has been reported as the foundation to building skills and confidence to undertake AMS [17]. Thus, trained pharmacists or infectious disease physicians, or both, can be utilised to facilitate GP-targeted AMS training programs. Peñalva et al. [18] demonstrated long-tern effectiveness and sustainability of multimodal AMS programs, including individual educational interviews in improving antibiotic use in primary care.

Our survey found that GPs who completed the NPS antimicrobial modules [10] had increased awareness of AMS and better uptake of AMS strategies. There is still a significant proportion of GPs who did not complete these courses or were not aware of it. Thus, dissemination of free educational modules and guidelines could be an effective approach to improving knowledge and confidence for optimal antimicrobial prescribing by Australian GPs. Van Katwyk et al. [19] identified 94 existing educational programs and modules on AMS/AMR globally but gaps exist in the provision of accredited training for current prescribers including GPs. In this regard, Australian stakeholders should create resource sharing platforms to increase accessibility and usability of AMS resources by GPs.

In the current study, there was homogeneity in the responses about the use of evidence-based AMS strategies across Australia. Most participants used Therapeutic Guidelines but strongly supported the integration of eTG with prescribing software. Higher uptake of delayed-prescribing strategy among Australian GPs was comparable with other developed country settings [20,21], though the impact of delayed-prescribing is dubious in reducing antibiotic consumption by patients. A survey in Australia by Avent et al. [22] showed that 40% of surveyed CPs (48/120) would dispense the delayed antibiotic prescription(s) within 24 h of the prescription being written by GPs. Thus, an effective GP–CP collaboration model is required to facilitate the implementation of delayed-prescribing approach in Australia. 

The observed poor adoption (10–20%) of point-of-care tests, patient information leaflets, peer- prescribing reports and provision of audit-feedback interventions likely reflects the lack of AMS implementation resources, system structures and facilities in general practices that was also backed up by qualitative data. Whilst the evidence of effectiveness and accessibility of GPs to diagnostic tests such as a rapid antigen detection test (RADT) to diagnose streptococcal pharyngitis, bronchitis and tonsillitis are increasing in other settings [14,15], the use of these tests remains low in Australian general practices. In summary, provision of using point-of-care tests, sustained monitoring of GPs’ antimicrobial prescriptions and post-prescription reviews by pharmacists could be experimented and tailored to improve guideline-adherent antimicrobial use in Australia. Stakeholders in the UK have also prioritised these interventions as the most promising and feasible to improve AMS in primary care [23].

The majority of GPs supported a policy that better leverage a GP–pharmacist collaboration to improve the prescribing of antimicrobials. This receptive attitude is important for developing a model to implement AMS by increased collaboration [12,13]. Indeed, the success of any GP–pharmacist collaborative model will also require a few existing challenges to be resolved. The challenges identified were isolated practice dynamics, poor communication structures, inter-professional trust and dependencies and the perceptions of some GPs that pharmacists are just the “dispensers”. Consistently, CPs’ attitudes towards this collaboration model in AMS could be worthy of further investigation.

Most GPs were receptive to a policy that limits the selected antimicrobials for certain clinical conditions. A European study has demonstrated an association between antibiotic consumption and number of antibiotics available in community settings [24]. For instance, Sweden and France have a total of 23 and 50 antibiotics available in the community, respectively [25,26]. Arguably, a policy initiative to restrict prescribing of some broad-spectrum antibiotics for certain clinical conditions might influence antimicrobial prescribing practice in Australian general practice.

In the current study, the respondents were non-receptive to strict policies on mandatory documentation of clinical indications in the clinical records. This is concerning because reporting of clinical indications has an association with the type of antibiotics that GPs choose to prescribe [27] and it’s non-reporting is a potential barrier to implementing post-prescription audits to assess AMS performance.

Our study has strengths and limitations. Our study was the first and largest nationwide AMS survey of Australian GPs. Although most surveys regarding AMS have been conducted in a hospital context [28,29], our study sheds light on primary care AMS including the issues of GP–pharmacist team-based implementation of AMS. Our survey tool achieved good reliability for international use with contextual validation. While our study response rate appears relatively low, it exceeds the published usual response rate of Australian GPs [30,31]. The distribution of surveyed GPs by gender and states was aligned with Australian census report though tendency of female GPs to participate in surveys is higher in general [32]. As participation in this survey was voluntary, GPs interested in AMS could have responded to the survey. This might lead to an overestimation of our findings. To improve the response rate, the future survey might consider some other strategies; requesting GPs to recommend other peers to participate, using social media interactions and increased networking but with scientific validity.

## 3. Materials and Methods

A published guideline [33] was used to conduct and report this survey study. We undertook a paper-based survey of a nationally representative sample of Australian GPs between January and May of 2019.

### 3.1. Development of the Survey Tool

Details of the survey development process are given in the supplementary materials (Figure S1). The survey questions were developed based on the reviewed literature [34,35,36,37,38,39,40] and validation by a panel of AMS experts through consensual approaches. AMS experts from the National Centre for Antimicrobial Stewardship (NCAS) together with GP researchers at the Department of General Practice of Monash University worked collaboratively to develop the survey tool. The panel of AMS experts involved an infectious disease physician (K.T.), academic GP (D.M.) and academic pharmacist (D.C.M.K.), who have extensive expertise in AMS implementation in Australia.

### 3.2. Description of Survey Tool

The 36-items survey tool (Table S8) consisted of 34 quantitative items divided into five sections (A–E); demographics (A), perceived awareness of AMS (B), current AMS practices (C), GP–pharmacist collaboration (D) and future improvement strategies (E) related to AMS. Two open-ended questions were related to the barriers and facilitators to improving AMS. The agreement scales used were the same as other validated surveys found from the literature search (S1). The survey tool used an agreement scale for 19 items (five-point Likert scale; 5 = strongly agree to 1 = strongly disagree) and for eight items (five-point Likert scale; 5 = always to 1= never). The survey tool was piloted amongst seven currently practicing GPs, and their positive feedback ensured generalisability.

### 3.3. Sampling Strategy

We required a sample size of 381 GPs to be able to derive statistically significant results [41]. We used the Australasian Medical Publishing Company (AmpCo) database [42] to select GPs with the national representativeness. GPs were first stratified by their practice location across Australia (six states: NSW, VIC, QLD, SA, WA, TAS and two territories: ACT, and NT) and then randomly selected using probability proportionate to GPs’ size in each state and territory.

### 3.4. Survey Deployment

Upon ethics approval, we reached 2500 GPs (NSW = 796, VIC = 552, QLD = 535, SA = 210, TAS = 75, ACT = 42 and NT = 24) with a package comprising of an invitation letter, explanatory statement, the survey questionnaire and a reply-paid envelope with two reminders via mail. Participating GPs were eligible to participate in a ‘lucky’ draw to win one of four $100 gift vouchers.

### 3.5. Data Analysis

We manually entered our data into a database and used a double manual data entry method [43] to clean the data. We compared the demographic characteristics of our sample to the population of GPs [44] in Australia where analysable using Χ^2^ tests. The five-point agreement responses were collapsed into three categories: (I) agree, neutral and disagree and (II) always/often, occasionally and rare/never. We calculated the percentage, mean and median for the categorical variables. An independent sample Mann-Whitney U test and Kruskal-Wallis test were performed for categorical variables. Logistic regression was done to identify the factors associated with the poor uptake of AMS strategies after deleting the missing data on a few items. The reliability and factor analysis were done using Cronbach’s alpha and principle component method for extraction, respectively. Eigenvalue (>1.0) statistics and scree plots were used to describe the finding of factor analyses. We used IBM SPSS statistic version 24 (SPSS) and Microsoft excel for data analysis. Qualitative data were analysed by using a framework of human factor engineering model, Systems Engineering Initiative for Patient Safety (SEIPS 2.0) [45,46].

## 4. Conclusions

Australian GPs are aware of AMS, but most do not routinely adopt known evidence-based AMS strategies. The majority of GPs hold positive attitudes towards working collaboratively with pharmacists in implementing AMS. Multiple barriers exist that hinder GPs’ ability to undertake AMS and collaborate with pharmacists to optimally prescribe antimicrobials. There are several opportunities to provide improved training of GPs around AMS, better access to stewardship resources and establish models of GP–pharmacist collaboration to support judicious antimicrobial prescribing in general practices. Future investigations are required from stakeholder’s perspectives to examine the feasibility of implementing the GP–pharmacist collaborative AMS strategies in Australian primary care.

## Figures and Tables

**Table 1:** Demographics of respondent general practitioners (GPs).

Demographics	Frequency (*n*)	Valid %	Australian GPs (*n* = 34,606)	Chi Square P Value
**Sex (*n =* 381)**				
Male	195	51.1		
Female	186	48.8	44.7	<0.109
**Education (*n =* 384)**				
B. Med science	4	1.0	-	
MBBS	305	79.4	-	
MD	31	8.0	-	
Masters	39	10.1	-	
PhD	5	1.3	-	
**Years of practice (*n =* 385)**				
≤5	20	5.2		
6–10	43	11.1		
>10	322	83.6		
**Current practice location (*n =* 384)**				
Metro	234	60.9	68.2	<0.0023
Regional	74	19.2	28.0	<0.0001
Rural	62	16.1	-	
Remote	14	3.6	3.9	<0.76
**State of work (*n =* 385)**				
New South Wales (NSW)	104	27.0	30.6	<0.127
Victoria (VIC)	105	27.2	24.1	<0.157
Queensland (QLD)	73	18.9	21.7	<0.184
Australian Capital Territory (ACT)	5	1.2	1.5	< 0.63
South Australia (SA)	39	10.1	7.8	<0.094
Western Australia (WA)	36	9.3	10.2	<0.561
Tasmania (TAS)	18	4.6	2.6	<0.014
Northern Territory (NT)	5	1.3	1.5	<0.75
**Medical training (*n =* 385)**				
Outside Australia	124	32.2	-	
Inside Australia	261	67.7	-	
**Completion of the National Prescribing Service’s (NPS’) antimicrobial prescribing course (*n =* 383)**				
Yes	105	27.4	-	
No	200	52.2	-	
Not aware	78	20.3	-	

“-” data unavailable for comparison.

**Table 2:** Major barriers and facilitators to improving AMS by Australian GPs.

Factors	Major Barriers	Major Facilitators
**Person**	Patient level: Patient expectations, lack of awareness regarding the risk of antibiotic use, late presentation of patients despite severe symptoms, desire for a quick recovery and poor health literacy. GP-level: GPs’ perception: “pharmacists are just a dispenser”, and “pharmacists have no adequate knowledge to educate GPs in AMS” Older GPs. Old habit of antibiotic prescribing. Inertia to change prescribing. Pharmacist level: conflict of interest to recommending antimicrobials, ignorance of a patient’s clinical records.	GPs’ willingness to follow AMS guidance AMS training. Confidence to not to prescribe antibiotics. Patients’ awareness and trust on doctors.
**Tools and technology**	No protocol that defines AMS tasks in practice. Lack of access and usability of Therapeutic Guidelines (TG) (Cost, broad recommendations Trustworthiness for some clinical conditions). IT facilities. Limited point-of- care testing facilities.	eTG (electronic Therapeutic Guidelines). Point-of-care tests. Clear guidelines on AMS task. Telehealth technologies. Patient communication tools.
**Organisation**	Lack of access to Infectious Disease physicians, pharmacists and microbiological services. Legal system for delayed prescribing. Delayed access to diagnostic reports (e.g., Antibiotic sensitivity, culture test). Lack of provision of AMS training. No monitoring and follow up of AMS related task.	AMS training programs.Access to infectious disease physicians, microbiologists and pharmacists. Weekly practice meeting for discussing AMS strategies. Improved “My Health Records”. Antimicrobial prescribing audit tools. Rapid testing results of antibiotic sensitivity. NPS-led visits and academic detailing.
**Task**	Time constrain to do AMS task and consulting with pharmacists	Increasing AMS staff time.Longer consultation time. Promoting delayed prescribing. Shared decision-making approach.
**Physical environment**	Information leaflets for educating patients	Patient education leaflets and posters. NPS handouts for treating infections.
**External environment**	Incentives. Funding model for AMS implementation. Extended validity of repeat antimicrobial prescriptions.	Media campaigns. GP–pharmacist group meetings. GP–pharmacy practice agreement. Policy limiting some broad-spectrum antibiotic prescription. Incentives for a longer consultation. Policy restricting repeat antimicrobial prescriptions.

## Supplementary Materials

The following are available online at https://www.mdpi.com/2079-6382/9/6/310/s1. Figure S1: Literature search and survey tool development, Table S1: Frequency distribution of responses, mean and median of AMS Awareness, Table S2: Frequency distribution of responses, mean and median for uptake of AMS strategies, Table S3: Frequency distribution of responses, mean and median of the attitudes towards GP–pharmacist collaboration in AMS, Table S4: Frequency distribution of responses, mean and median of the attitude towards future AMS improvement strategies, Table S5: Variation in GPs’ awareness, uptake and attitudes towards GP–pharmacist collaboration concerning AMS by characteristics, Table S6: Logistic regression analysis of AMS awareness, uptake of AMS strategies and GP–pharmacist collaboration by demographic variables, Table S7: Factor analysis of AMS practices, Table S8: Survey Instrument, Figure S2: Principal component analysis.
